# Ablação por Cateter é Superior a Drogas Antiarrítmicas como Tratamento de primeira linha para Fibrilação Atrial: uma Revisão Sistemática e Metanálise

**DOI:** 10.36660/abc.20210477

**Published:** 2022-04-25

**Authors:** Rhanderson Cardoso, Gustavo B. Justino, Fabrissio P. Graffunder, Leticia Benevides, Leonardo Knijnik, Luana M.F. Sanchez, Andre d’Avila

**Affiliations:** 1 Heart and Vascular Center Brigham and Women’s Hospital Harvard Medical School Boston MA EUA Heart and Vascular Center, Brigham and Women’s Hospital, Harvard Medical School, Boston, MA – EUA; 2 Departamento de Medicina Universidade Federal de Santa Catarina Florianópolis SC Brasil Departamento de Medicina, Universidade Federal de Santa Catarina, Florianópolis, SC – Brasil; 3 Departamento de Medicina Universidade Federal do Ceará Fortaleza CE Brasil Departamento de Medicina, Universidade Federal do Ceará, Fortaleza, CE – Brasil; 4 Departamento de Medicina University of Miami Miami EUA Departamento de Medicina, University of Miami, Miami – EUA; 5 Departamento de Medicina Universidade Mauricio de Nassau Recife PE Brasil Departamento de Medicina, Universidade Mauricio de Nassau, Recife, PE – Brasil; 6 Serviço de Arritmia Cardíaca Hospital SOS Cárdio Florianópolis SC Brasil Serviço de Arritmia Cardíaca, Hospital SOS Cárdio, Florianópolis, SC – Brasil; 7 Harvard-Thorndike Electrophysiology Institute Beth Israel Deaconess Medical Center Harvard Medical School Boston MA EUA Harvard-Thorndike Electrophysiology Institute, Beth Israel Deaconess Medical Center, Harvard Medical School, Boston, MA – EUA

**Keywords:** Ablação por Cateter, Antiarrítmicos, Fibrilação Atrial

## Abstract

**Fundamento:**

A ablação por cateter é uma terapia bem estabelecida para controle do ritmo cardíaco em pacientes refratários ou intolerantes a drogas antiarrítmicas (DAA). Porém, a eficácia desse procedimento comparada à de DAA como estratégia de primeira linha no controle do ritmo cardíaco na fibrilação atrial é menos conhecida.

**Objetivos:**

Conduzir uma revisão sistemática e metanálise da ablação por cateter vs. DAA em pacientes sem nenhum tratamento prévio para controle do ritmo.

**Métodos:**

Buscamos, nos bancos de dados do PubMed, EMBASE, e Cochrane, ensaios randomizados controlados que compararam ablação por cateter com DAA para controle do ritmo cardíaco em pacientes com FA sintomática e descreveram os seguintes desfechos: (1) recorrência de taquiarritmia atrial (TA); (2) FA sintomática; (3) internações hospitalares; e (4) bradicardia sintomática. A heterogeneidade foi avaliada por estatística I^2^. Valores de p menores que 0,05 foram considerados estatisticamente significativos.

**Resultados:**

Incluímos cinco ensaios com 994 pacientes, dos quais 502 (50,5%) foram submetidos à ablação por cateter. O período médio de acompanhamento foi de um a cinco anos. Recorrências de TA (OR 0,36; IC95% 0,25-0,52; p<0,001) e de FA sintomática (OR 0,32; IC95% 0,18-0,57; p<0,001), e internações hospitalares (OR 0,25; IC95% 0,15-0,42; p<0,001) foram menos frequentes nos pacientes tratados com ablação por cateter que naqueles tratados com DAA. Bradicardia sintomática não foi diferente entre os grupos (OR 0,55; IC95% 0,18-1,65; p=0,28). Derrame ou tamponamento pericárdico significativo ocorreu em oito dos 464 (1,7%) pacientes no grupo submetido à ablação.

**Conclusão:**

Esses achados sugerem maior eficácia da ablação por cateter que das DAA como estratégia inicial de controle do ritmo cardíaco em pacientes com DA sintomática.

## Introdução

A fibrilação atrial (FA) é uma condição de alta prevalência, que afeta aproximadamente 50 milhões de pessoas no mundo.^1 , 2^ A prevalência global da FA continua a aumentar, provavelmente pelo envelhecimento populacional e crescente prevalência da obesidade e doença cardiometabólica. Somente nos EUA, mais de 12 milhões de pessoas podem desenvolver FA até o ano de 2030.^1 , 2^ O diagnóstico e a carga da FA estão associados com aumento na mortalidade, em eventos cerebrovasculares, insuficiência cardíaca, e internações hospitalares.^3 , 4^ No entanto, uma melhora na sobrevida, na qualidade de vida, e na sobrevida livre de eventos não fatais pode ser alcançada com estratégias efetivas de anticoagulação, controle do ritmo e/ou frequência cardíaca em pacientes selecionados.^3 - 5^

A terapia com drogas antiarrítmicas (DAA) e a ablação por cateter com isolamento da veia pulmonar são duas opções terapêuticas bem definidas para o controle do ritmo cardíaco, quando se deseja a manutenção do ritmo cardíaco. Contudo, ambas as estratégias apresentam deficiências, incluindo uma eficácia limitada. A terapia com DAA pode causar efeitos colaterais, interações medicamentosas, e arritmias ventriculares, e a ablação por cateter é um procedimento invasivo, com potencial para raras, mas sérias complicações. Nas diretrizes mais recentes de várias sociedades europeias e norte-americanas, a ablação por cateter é recomendada como indicação classe I para pacientes sem sucesso com DAA, embora seu uso como terapia de primeira linha seja menos recomendado.^3 , 4^

Recentemente, dois ensaios grandes, randomizados, investigaram o papel da ablação por cateter como uma terapia de primeira linha para o controle do ritmo em pacientes com FA sintomática.^6 , 7^ Esses estudos aumentaram substancialmente a população de pacientes randomizados para receberem ablação por cateter ou DAA como estratégia de primeira linha para controle do ritmo. Assim, nosso objetivo foi realizar uma revisão sistemática e metanálise comparando essas duas estratégias em estudos randomizados, avaliando desfechos de eficácia em uma grande população, bem como examinar desfechos secundários, nos quais os estudos individuais possam ter menos força.

## Materiais e Métodos

### Critérios de elegibilidade e extração dos dados

Restringimos nossa análise de estudos que preenchessem todos os seguintes critérios de inclusão: (1) ensaios controlados randomizados (ECRs) de ablação por cateter vs. DAA; (2) inclusão de pacientes com FA sintomática que não receberam tratamento com DAA; e (3) análise de quaisquer dos seguintes desfechos de interesse – recorrência de taquicardia atrial, recorrência de FA sintomática, hospitalizações, bradicardia sintomática, e qualidade de vida. Os critérios de exclusão incluíram estudos não randomizados, ensaios incluindo pacientes submetidos previamente à ablação por cateter ou à terapia com DAA sem sucesso. Em caso de estudos com sobreposição de pacientes, foi incluído aquele com o maior número de pacientes. Não houve restrições quanto à inclusão com base no tamanho da população estudada.

Dois autores (G.B.J. e L.B.S.) extraíram os dados independentemente, seguindo critérios de busca e métodos de avaliação de qualidade pré-definidos. Discordâncias entre esses autores foram resolvidas por consenso entre três autores (R.C., G.B.J. e L.B.S.).

### Estratégia de busca

Realizamos uma busca sistemática no PubMed, EMBASE, e *Cochrane Central Register of Controlled Trials* . A busca foi conduzida sem restrições de data em dezembro de 2020 por estudos publicados somente em inglês. Os seguintes termos foram inseridos na busca: “atrial fibrillation” AND (“ablation” OR “radiofrequency” OR “cryoablation” OR “cryoballoon”) AND (“antiarrhythmic” OR “AAD” OR “amiodarone” OR “sotalol” OR “flecainide” OR “propafenone” OR “dofetilide”) AND (“first-line” OR “initial”). Além disso, as listas de referências de todos os estudos, metanálises e revisões incluídos foram revisadas manualmente.

### Avaliação da qualidade

O risco de viés e a avaliação da qualidade de cada estudo foram analisados com a ferramenta do Cochrane Collaboration para avaliação do risco de viés em estudos randomizados.^8^ Cada estudo recebeu um escore de “alto risco”, “baixo risco” ou “risco incerto” para cada um dos cinco domínios: viés de seleção, desempenho, detecção, perda (de pacientes) e relato. Foram construídos gráficos de funil dos pesos individuais dos estudos em relação às estimativas para avaliar a evidência de viés de publicação.

### Análise estatística

A revisão sistemática e a metanálise foram realizadas de acordo com a ferramenta de avaliação de risco de viés do Cochrane Collaboration e das recomendações do *Preferred Reporting Items for Systematic Reviews and Meta-Analysis* (PRISMA).^9^ Odds ratios (OR) com intervalo de confiança de 95% foram calculados para comparar a incidência de desfechos binários entre os dois braços de tratamento. Usamos o teste Q de Cochran e estatística I^2^ para avaliar heterogeneidade. Os desfechos foram considerados de baixa heterogeneidade se p>0,10 e I^2^< 25%. Usamos um modelo de efeitos fixos para desfechos com I^2^< 25% (baixa heterogeneidade). Nos desfechos com alta heterogeneidade, estimativas agrupadas foram computadas com modelo de efeitos aleatórios de DerSimonian e Laird. Valores de p < 0,05 foram considerados estatisticamente significativos. Análises estatísticas foram realizadas com o programa Review Manager 5.4 (Nordic Cochrane Centre, The Cochrane Collaboration, Copenhague, Dinamarca).

## Resultados

**Figura 1:**
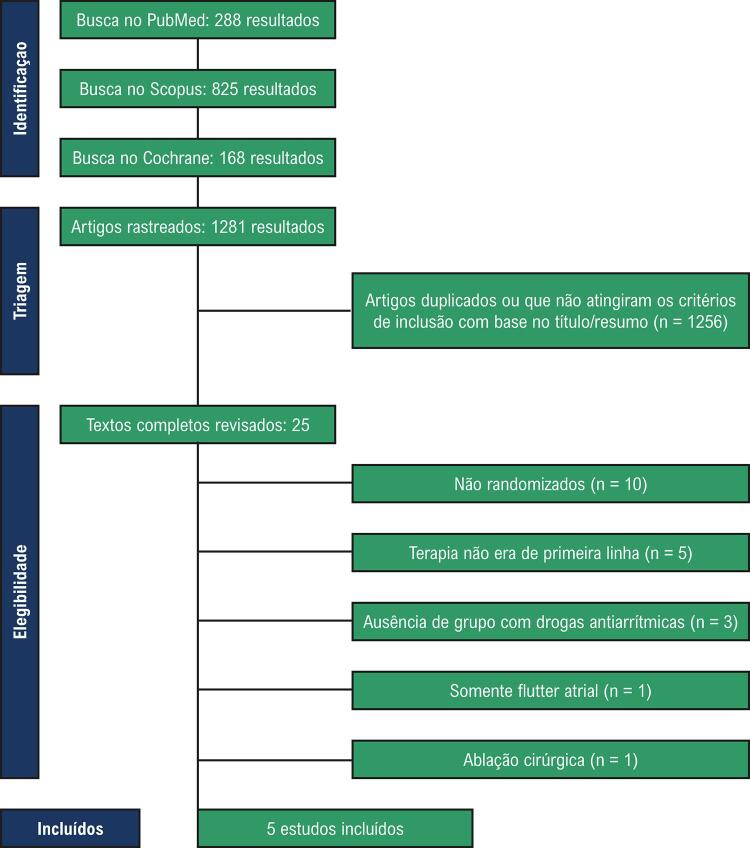
– Fluxograma PRISMA da triagem e seleção dos estudos

Como detalhado na Figura 1 , foram identificados 1281 estudos pela estratégia de busca nos bancos de dados e busca manual por referências de revisões e metanálises pertinentes. Após a exclusão dos artigos em duplicata e estudos não relacionados, 25 artigos foram revisados quanto aos critérios de inclusão e exclusão. Foram incluídos cinco estudos, com total de 994 pacientes, dos quais 502 (50,5%) foram submetidos à ablação por cateter.^6 , 7 , 10 - 12^ As características dos pacientes estão apresentadas na Tabela 1 . Os estudos foram homogêneos quanto à técnica de ablação, monitoramento de taquiarritmia atrial (TA) recorrente, e tempo de acompanhamento, que variou de um a cinco anos.

**Tabela 1:** – Características basais dos estudos incluídos

	Número de pacientes	Homens, n (%)	Idade média (anos)	Técnica de ablação por cateter	Terapia com DAA	Monitoramento da TA	FA paroxística, n (%)	Tempo médio do diagnóstico da FA (meses)	FEVE médio (%)	Seguimento (anos)
RAAFT-1 2005	67	NA	CA: 53 AAD: 54	RF	Flecainida, 77% Sotalol, 23% Nenhuma droga antiarrítmica relatada no grupo da ablação	Holter 24 horas antes da alta, e em 3, 6 e 12 meses após	AC: 32 (97) DAA: 35 (95)	5	AC: 53 DAA: 54	1
MANTRA-PAF 2012	294	CA: 100 (68) AAD: 106 (72)	CA: 56 AAD: 54	RF	Drogas classe IC de preferência; segunda linha classe III; DAA permitidas durante período de estabilização nos pacientes submetidos à ablação	Holter 7 dias em 3, 6, 12, 18 e 24 meses	AC: 146 (100) DAA: 148 (100)	NA	FEVE >60%: 237 (80%)	5*
RAAFT-2 2014	127	CA: 51 (77.3) AAD: 45 (73.8)	CA: 56 AAD: 54	RF	Durante período de estabilização (90 dias): Flecainida, 69%; propafenona 25%; dronedarona 3%. DAA permitidas no grupo da ablação	ECG, Holter, Monitor transtelefônico, ou traçado do ritmo cardíaco	AC: 65 (98) DAA: 59 (97)	NA	A:C 61 DAA: 61	2
STOP-AF 2020	203	CA: 63 (61) AAD: 57 (58)	CA: 60 AAD: 62	Crioablação	No grupo DAA: flecainida 60%; propafenoa 7%; dronedarona 12%; sotalol 7%; amiodarona 2%. No grupo ablação, DAA durante período de estabilização (80 dias)	ECG conduzido no basal, em 1, 3, 6, e 12 meses; monitoramento por telefone ativado pelo paciente semanalmente e na presença de sintomas em 3-12 meses; monitoramento ambulatorial 24h em 6 e 12 meses	AC: 104 (100) DAA: 99 (100)	15.6	AC: 61 DAA: 61	1
EARLY-AF 2020	303	AC: 112 (72,7) DAA: 102 (68,5)	AC: 58 DAA: 59	Crioablação	Flecainida 76%; propafenona 5%; sotalol 15%; dronedarona 3%; DAA no período de estabilização	Monitor cardíaco implantável; transmissão manual semanalmente; visitas em 3, 6 e 12 meses	AC: 147 (95) DAA: 140 (94)	1	AC: 60 DAA: 60	1

*Valores p < 0,05 foram considerados estatisticamente significativos em todos os estudos; ‡hipertensão e doença cardíaca estrutural; DAA: drogas antiarrítmicas; FA: fibrilação atrial; TA: taquiarritmia atrial; AC: ablação por cateter; FEVE: fração de ejeção do ventrículo esquerdo; ND: não disponível; RF: radiofrequência.*

**Figura 2:**
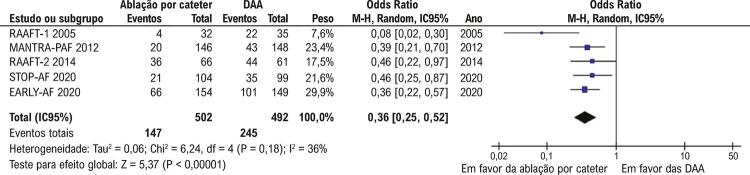
– Recorrências de taquiarritmia atrial foram significativamente menos comum com ablação por cateter que com terapia com drogas antiarrítmicas (p<0,001). DAA: drogas antiarrítmicas.

**Figura 3:**
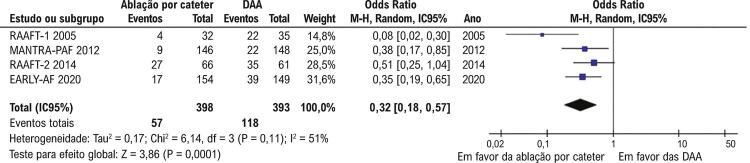
– Recorrências de fibrilação atrial sintomática foram significativamente menos comum com ablação por cateter que com terapia com drogas antiarrítmicas (p<0,001). DAA: drogas antiarrítmicas.

**Figura 4:**
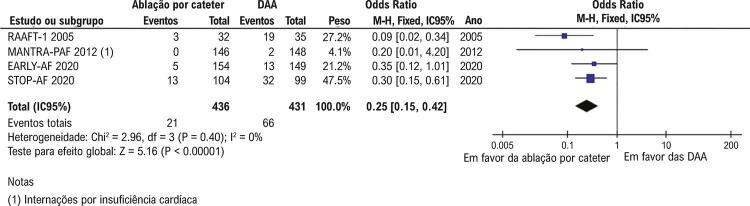
– Internações hospitalares foram significativamente menos comum com ablação por cateter que com terapia com DAA (p<0,001). DAA: drogas antiarrítmicas.

A recorrência de TA foi significativamente menos frequente nos pacientes tratados com ablação por cateter (147/502; 29,2%) em comparação à DAA (245/492; 49,8%) (OR 0,36; IC95% 0,25-0,52; p<0,001; Figura 2 ). Além disso, a recorrência de FA sintomática também foi menor nos pacientes que randomizados para receberem ablação por cateter (57/398; 14,3%) em comparação aos que receberam DAAs (118/393; 30%) (OR 0,32; IC95% 0,18-0,57; p<0,001; Figura 3 ). As internações hospitalares também foram menos frequentes no grupo submetido à ablação (21/436; 4,8% vs. 66/431; 15,3%) (OR 0,25; IC95% 0,15-0,42; p<0,001; Figura 4 ).

**Figura 5:**
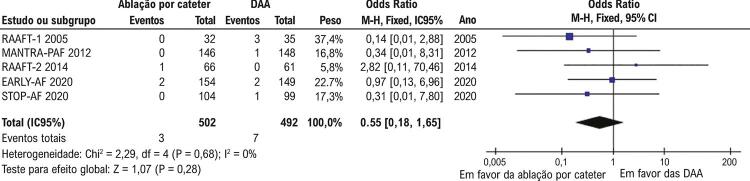
– Incidência de bradicardia sintomática foi rara e similar entre os grupos (p=0,28). DAA: drogas antiarrítmicas.

Em relação aos desfechos de segurança, bradicardia sintomática (OR 0,55; 95% CI 0.18-1.65; p=0.28; I^2^=0%; Figura 5 ) não foi significativamente diferente entre pacientes tratados com ablação por cateter (3/502; 0,6%) e terapia com DAA (7/492; 1,4%). Derrame ou tamponamento pericárdico de significância clínica ocorreu em oito dos 464 pacientes no grupo de ablação por cateter (1,7%).

A Tabela 1 descreve a apreciação da qualidade de cada ECR. Todos os estudos foram considerados em risco de viés de desempenho, dada a impossibilidade do delineamento duplo cego (paciente e investigador) nos ensaios, e considerados de baixo risco quanto aos demais vieses. Análises de sensibilidade foram realizadas pela exclusão sistemática de cada estudo das estimativas agrupadas. Após a remoção de cada estudo individual, os resultados para recorrência de TAs, FA sintomática, internações hospitalares, e bradicardia sintomática não se modificaram. Apesar do pequeno número de estudos, não houve evidência definitiva de viés de publicação nos gráficos de funil ( Figura 1 Suplementar ).

## Discussão

Nesta revisão sistemática e metanálise de cinco estudos e 994 pacientes, nós comparamos o procedimento de ablação por cateter e DAA como terapia de primeira linha para controle do ritmo cardíaco em pacientes com FA. Os principais achados foram: (1) a incidência de FA recorrente sintomática durante um período de um a cinco anos de seguimento foi aproximadamente a metade no grupo submetido à ablação em comparação ao grupo tratado com DAA (14,3% vs. 30,0%, respectivamente; OR 0,32; p<0,001); (2) essa diferença também foi estatisticamente significativa para redução de TAs, em favor do procedimento de ablação (OR 0,36; p<0,001); e (3) a taxa de hospitalização foi três vezes menor nos pacientes submetidos à ablação (4,8% vs. 14,3%).

A ablação por cateter mostrou-se superior ao aumento na terapia com DAA para controle do ritmo em pacientes com FA recorrente apesar de uma tentativa anterior de terapia com DAA ou ablação prévia. No estudo CABANA, 2.204 pacientes com FA sintomática em uso atual ou recente de ≥ 1 droga antiarritmmica foram randomizados para ablação de FA por cateter ou terapia antiarritmica. Na análise por intenção de tratar, durante um período mediano de seguimento ode 48,5 meses, a recorrência de FA ocorreu em 49,9% dos pacientes randomizados para ablação por cateter e 69,5% naqueles tratados com DAA (HR 0,52; IC95% 0,45-0,60; p<0,001). O desfecho primário de morte, incapacidade associada a acidente vascular cerebral, sangramento grave ou ataque cardíaco não foi significativamente diferente entre ablação e terapia com DAA (8% vs. 9,2%, respectivamente; HR 0,86; p=0,30).^13^

Apesar dos resultados desapontadores de mortalidade de desfechos vasculares, não há dúvida de que o CABANA e outros ensaios demonstraram uma maior eficácia da ablação por cateter em comparação à terapia com DAA isolada em pacientes previamente tratados com esses medicamentos para controle do ritmo, sem sucesso.^13 - 16^ No entanto, existe um interesse renovado em um controle precoce efetivo do ritmo na história natural da FA. De fato, a noção de que FA gera FA, devido à fibrose atrial e remodelamento adverso, é bem conhecida há quase três décadas.^17 , 18^ No ensaio recentemente publicado *Treatment of Atrial Fibrillation for Stroke Prevention Trial* (EAST-AFNET 4), 2789 pacientes com FA diagnosticados há menos de 12 meses (27% com FA persistente) foram randomizados para controle precoce do ritmo com ablação por cateter (8%) ou DAA (87%) ou ao tratamento convencional com controle da frequência e ritmo cardíacos para sintomas refratários. Durante um período mediano de 5,1 anos, observou-se uma redução significativa no desfecho primário de morte cardiovascular, acidente vascular cerebral, ou internações por insuficiência cardíaca ou síndrome coronária aguda no grupo submetido ao controle precoce do ritmo cardíaco (3,9 por 100 pessoas-ano) em comparação ao tratamento convencional (5,0 por 100 pessoas-ano) (HR 0,79; IC96% 0,66-0,94; p=0,005).^19^

O controle precoce do ritmo com DAA, contudo, é limitado pela baixa eficácia da terapia medicamentosa isolada. Uma revisão sistemática e metanálise recente da Cochrane Collaboration avaliaram a eficácia e a segurança das DAAs em 59 ECRs com 20 981 participantes, incluindo tanto FA paroxística como FA persistente. Durante um período médio de acompanhamento de 10,2 meses, a FA foi recorrente em 43-67% dos pacientes tratados com DAAs.^20^ A eficácia limitada da terapia com DAA é bem evidente ao considerar a alta taxa de cruzamento de pacientes recebendo DAA para o tratamento por ablação nos ensaios randomizados. No ensaio “ *STOP AF First: Cryoballoon Catheter Ablation in Antiarrhythmic Drug Naïve Paroxysmal Atrial Fibrillation* ”, um terço dos pacientes no grupo DAA foi submetido à ablação por cateter devido a efeitos colaterais da terapia medicamentosa ou FA recorrente.^6^ No estudo CABANA, 27,5% dos pacientes no grupo DAA passou para o grupo da ablação durante o seguimento.^13^

Ensaios mais antigos comparando a eficácia da ablação por cateter com a terapia com DAA em pacientes que não receberam nenhum tipo de tratamento para controle do ritmo cardíaco foram limitados quanto ao tamanho amostral.^10 , 12^ No conjunto, esses estudos não demonstraram de maneira conclusiva uma superioridade da ablação por cateter em relação à terapia com DAA.^10 - 12^ Uma metanálise desses ensaios mostrou uma menor sobrevida livre de recorrência de FA nos pacientes submetidos à ablação em comparação à terapia com [razão de risco (RR) 0,63; IC95% 0,44-0,92; p=0,02]. No entanto, a taxa de recorrências de FA sintomática não foi significativamente diferente entre os grupos (RR 0,57; IC95% 0,30-1,08; p=0.09).^21^ Assim, o STOP AF First e o *Cryoablation or Drug Therapy for Initial Treatment of Atrial Fibrillation* (EARLY-AF) foram desenvolvidos para investigar o papel da ablação por cateter como uma estratégia de primeira linha no controle do ritmo cardíaco.

Nossos achados fornecem um entendimento mais preciso do efeito do tratamento reunindo um grande número de pacientes que foram divididos aleatoriamente para ablação por cateter ou terapia com DAA. A magnitude do efeito em favor da ablação por cateter foi substancial. A redução absoluta na frequência de TA e FA sintomática com ablação por cateter foi de 20% e 15%, respectivamente. Ao considerar esses achados, os desfechos de segurança da ablação por cateter não devem ser negligenciados no direcionamento das tomadas de decisão. A incidência agrupada de derrame e/ou tamponamento pericárdico nesses estudos foi de 1,7%. Em uma metanálise de aproximadamente 9.000 pacientes submetidos à crioablação ou à ablação por radiofrequência, a incidência de tamponamento pericárdico foi de 1,1%. Paralisia do nervo frênico ocorreu em 1,6% dos pacientes submetidos à crioablação, mas a maioria dos casos foi revertida durante o acompanhamento em curto prazo.^22^

Como se observa na Tabela 1 , as técnicas de ablação foram heterogêneas entre os estudos. Nos três estudos mais antigos usou-se ablação por radiofrequência, enquanto nos estudos mais recentes – STOP-AF e EARLY-AF – usou-se a crioablação.^6 , 7 , 10 - 12^ Embora as técnicas tenham importantes diferenças nas curvas de aprendizagem e desfechos de segurança, os ensaios FIRE e ICE^23^ e uma metanálise^22^ mostraram eficácia similar entre as duas técnicas. Mais importante, a tecnologia de radiofrequência melhorou substancialmente nos últimos anos, particularmente com o desenvolvimento de sensores de força de contato, os quais não foram usados no presente estudo. Uma metanálise com 22 estudos mostrou que a ablação por contato guiada por força de contato reduziu consideravelmente o tempo de procedimento, e melhorou a taxa de sobrevida livre de FA em 12%.^24^ Não se sabe se o uso de tecnologias mais novas para a ablação por radiofrequência modificaria a eficácia comparativa da ablação por cateter vs. DAA no controle inicial de ritmo cardíaco na FA sintomática. Se sim, isso se traduziria em um efeito ainda mais favorável da ablação em relação à terapia com DAA.

Nosso estudo tem limitações. Primeiro, o acompanhamento em longo prazo, além de dois anos, somente foi realizado em dois dos cinco estudos avaliados. Segundo, a estratégia de monitoramento do ritmo foi heterogênea entre os estudos, conforme descrito na Tabela 1 , variando desde monitoramento periódico por Holter até monitoramento cardíaco contínuo. No entanto, análise de sensibilidade removendo um estudo por vez não alterou a significância das estimativas de eficácia. Terceiro, a ausência de dados individuais dos pacientes impediu uma avaliação mais detalhada dos desfechos, tais como tempo para recorrência de TA/FA. Por fim, o pequeno número de estudos impediu a realização de análises de subgrupos de diferentes técnicas de ablação por cateter. Contudo, uma metanálise anterior mostrou eficácia similar entre radiofrequência e crioablação por balão.^22^

## Conclusão

Em resumo, a ablação por cateter reduz significativamente a recorrência da TA e FA sintomática em comparação à terapia com DAA em pacientes sem nenhuma tentativa prévia de tratamento de controle do ritmo. Este estudo apresenta evidências em favor do procedimento de ablação por cateter como indicação classe I para o controle de ritmo cardíaco em pacientes com FA paroxística.

### Acordo de compartilhamento de dados

Uma vez que esta metanálise baseou-se em dados extraídos de pesquisas publicadas, todos os dados e materiais dos estudos estão disponíveis no domínio público. Os autores desta metanálise não tiveram acesso aos dados dos pacientes dos estudos analisados, e encorajamos pesquisadores interessados nesses dados que contatem o autor (para correspondência) de cada estudo.

## *Material suplementar

Para informação adicional, por favor,clique aqui


