# Supplementary material for: Ablação por Cateter é Superior a Drogas Antiarrítmicas como Tratamento de primeira linha para Fibrilação Atrial: uma Revisão Sistemática e Metanálise
Source: Arq Bras Cardiol. 2022 Apr 25;119(1):87–94. [Article in Portuguese] doi: 10.36660/abc.20210477 (PMC9352118; doi:10.36660/abc.20210477)

Supplementary Table 1. Critical appraisal of individual studies according to the Cochrane Collaboration's tool for assessing risk of bias in randomized trials.

Study	Selection bias	Performance bias	Detection bias	Attrition bias	Reporting bias
RAAFT-1 2005	Low	High	Unclear	Low	Low
MANTRA-PAF 2012	Low	High	Low	Low	Low
RAAFT-2 2014	Low	High	Low	Low	Low
STOP-AF 2020	Low	High	Unclear	Low	Low
EARLY-AF 2020	Low	High	Low	Low	Low

Supplementary Figure 1. Funnel plot for recurrences of atrial tachyarrhythmias (1A), symptomatic AF recurrences (1B), hospitalizations (1C), and symptomatic bradycardia (1D) showed no definitive evidence of publication bias. Sensitivity analyses excluding each study found no change in significance of results.

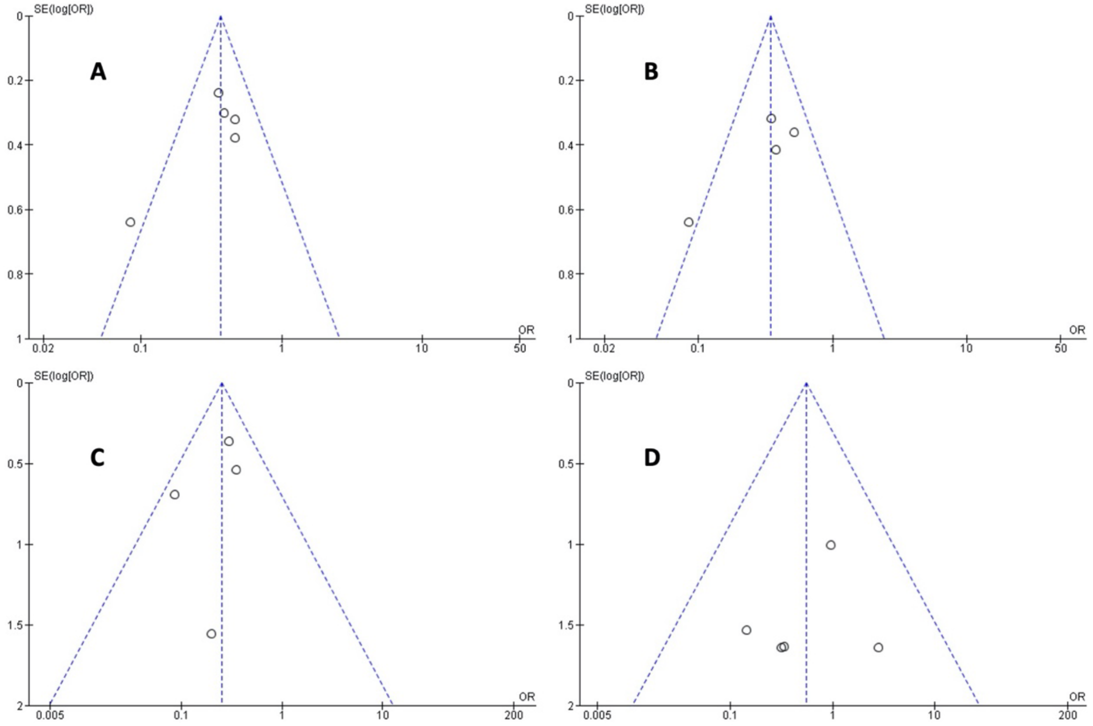

Supplement: Supplementary file 1 [file 2021-0477-supplementary.pdf]
