# Supplementary material for: Ablação por Cateter é Superior a Drogas Antiarrítmicas como Tratamento de primeira linha para Fibrilação Atrial: uma Revisão Sistemática e Metanálise
Source: Arq Bras Cardiol. 2022 Apr 25;119(1):87–94. [Article in Portuguese] doi: 10.36660/abc.20210477 (PMC9352118; doi:10.36660/abc.20210477)
Supplement: Translation

# Translation: Catheter Ablation is Superior to Antiarrhythmic Drugs as First-Line Treatment for Atrial Fibrillation: a Systematic Review and Meta-Analysis

**Authors:** Rhanderson Cardoso, Gustavo B. Justino, Fabrissio P. Graffunder, Leticia Benevides, Leonardo Knijnik, Luana M.F. Sanchez, Andre d’Avila

**Affiliations:** 1 EUA Heart and Vascular Center, Brigham and Women’s Hospital, Harvard Medical School, Boston, MA – EUA; 2 Brasil Departamento de Medicina, Universidade Federal de Santa Catarina, Florianópolis, SC – Brasil; 3 Brasil Departamento de Medicina, Universidade Federal do Ceará, Fortaleza, CE – Brasil; 4 EUA Departamento de Medicina, University of Miami, Miami – EUA; 5 Brasil Departamento de Medicina, Universidade Mauricio de Nassau, Recife, PE – Brasil; 6 Brasil Serviço de Arritmia Cardíaca, Hospital SOS Cárdio, Florianópolis, SC – Brasil; 7 EUA Harvard-Thorndike Electrophysiology Institute, Beth Israel Deaconess Medical Center, Harvard Medical School, Boston, MA – EUA

**Keywords:** Catheter Ablation, Anti-Arrhythmia Agents, Atrial Fibrillation

## Abstract

**Background:**

Catheter ablation is a well-established therapy for rhythm control in patients who are refractory or intolerant to anti-arrhythmic drugs (AAD). Less is known about the efficacy of catheter ablation compared with AAD as a first-line strategy for rhythm control in atrial fibrillation (AF).

**Objectives:**

We aimed to perform a systematic review and meta-analysis of catheter ablation vs. AAD in patients naïve to prior rhythm control therapies.

**Methods:**

PubMed, EMBASE, and Cochrane databases were searched for randomized controlled trials that compared catheter ablation to AAD for initial rhythm control in symptomatic AF and reported the outcomes of (1) recurrent atrial tachyarrhythmias (ATs); (2) symptomatic AF; (3) hospitalizations; and (4) symptomatic bradycardia. Heterogeneity was examined with I^2^statistics. P values of < 0.05 were considered statistically significant.

**Results:**

We included five trials with 994 patients, of whom 502 (50.5%) underwent catheter ablation. Mean follow-up ranged from one to five years. Recurrences of AT (OR 0.36; 95% CI 0.25-0.52; p<0.001) and symptomatic AF (OR 0.32; 95% CI 0.18-0.57; p<0.001), and hospitalizations (OR 0.25; 95% CI 0.15-0.42; p<0.001) were significantly less frequent in patients treated with catheter ablation compared with AAD. Symptomatic bradycardia was not significantly different between groups (OR 0.55; 95% CI 0.18-1.65; p=0.28). Significant pericardial effusions or tamponade occurred in eight of 464 (1.7%) patients in the catheter ablation group.

**Conclusion:**

These findings suggest that catheter ablation has superior efficacy to AAD as an initial rhythm control strategy in patients with symptomatic AF.

## Introduction

Atrial fibrillation (AF) is a highly prevalent condition, estimated to affect nearly 50 million people worldwide.^1 , 2^The global prevalence of AF continues to increase, likely related to population aging and the rising prevalence of obesity and cardiometabolic disease. In the US alone, more than 12 million individuals may have AF by 2030.^1 , 2^The diagnosis and burden of AF are associated with increased mortality, cerebrovascular events, heart failure, and hospitalizations.^3 , 4^However, improved survival, quality of life, and freedom from non-fatal events can be achieved with effective strategies of anticoagulation, heart rate control, and/or rhythm control in selected patients.^3 - 5^

Antiarrhythmic drug (AAD) therapy and catheter ablation with pulmonary vein isolation are two well-established options for rhythm control when maintenance of sinus rhythm is desirable. However, both strategies have drawbacks, including limited efficacy. AAD can lead to side effects, drug-drug interactions, and ventricular arrhythmias, and catheter ablation is an invasive procedure, with the potential for rare but serious complications. In the most recent multi-society guidelines from Europe and North America, catheter ablation is recommended as a class I indication for patients who fail a strategy of AAD, whereas its use as a first-line therapy is less recommended.^3 , 4^

Recently, two large, randomized trials have explored the role of catheter ablation as first-line therapy for rhythm control in patients with symptomatic AF.^6 , 7^These trials have greatly increased the population of randomized patients who underwent either catheter ablation or AAD as a first-line strategy for rhythm control. Therefore, we sought to perform a systematic review and meta-analysis comparing these two strategies in randomized studies, evaluating efficacy outcomes in a large population, as well as to examine secondary endpoints, for which the individual studies may be underpowered.

## Material and methods

### Eligibility criteria and data extraction

We restricted our analysis to studies that met all the following inclusion criteria: (1) randomized controlled trials (RCTs) of catheter ablation vs. AADs; (2) inclusion of patients with symptomatic AF who had not received any AAD treatment; and (3) analysis of any of the following outcomes of interest – recurrence of atrial tachyarrhythmias, recurrence of symptomatic AF, hospitalizations, symptomatic bradycardia, and quality of life. The exclusion criteria included non-randomized studies, and trials including patients who had previously failed catheter ablation or AAD therapy. In case of studies with overlapping patient populations, the study with the largest number of patients was included. There were no restrictions for inclusion based on the size of the study population.

Two authors (G.B.J. and L.B.S.) independently extracted data following pre-defined search criteria and quality assessment methods. Disagreements between these authors were resolved by consensus among three authors (R.C., G.B.J., and L.B.S.).

### Search strategy

We systematically searched PubMed, EMBASE, and the Cochrane Central Register of Controlled Trials. The search was conducted without date restrictions in December 2020 for studies published in English only. The following medical subject heading terms were included: “atrial fibrillation” AND (“ablation” OR “radiofrequency” OR “cryoablation” OR “cryoballoon”) AND (“antiarrhythmic” OR “AAD” OR “amiodarone” OR “sotalol” OR “flecainide” OR “propafenone” OR “dofetilide”) AND (“first-line” OR “initial”). In addition, the reference lists of all included studies, meta-analyses and reviews were manually searched.

### Quality assessment

Risk of bias and quality assessment of individual studies were analyzed with the Cochrane Collaboration’s tool for assessing risk of bias in randomized studies.^8^Each trial was given a score for “high risk”, “low risk”, or “unclear risk” in each of the five domains: selection, performance, detection, attrition, and reporting biases. Funnel plots of individual study weights against point estimates were used to check for evidence of publication bias.

### Statistical analysis

Systematic review and meta-analysis were performed according to the Cochrane Collaboration’s tool for assessing risk of bias and the Preferred Reporting Items for Systematic Reviews and Meta-Analysis (PRISMA) statement.^9^Odds ratios (OR) with 95% confidence intervals (CI) were computed to compare the incidence of binary endpoints between the two treatment arms. We used Cochran’s Q test and I^2^statistics to evaluate for heterogeneity. Endpoints were considered to have low heterogeneity if p > 0.10 and I^2^< 25%. We used a fixed-effect model for endpoints with I^2^< 25% (low heterogeneity). In outcomes with high heterogeneity, pooled estimates were computed with DerSimonian and Laird random-effects model. P values of < 0.05 were considered statistically significant. Statistical analyses were performed with Review Manager 5.4 (Nordic Cochrane Centre, The Cochrane Collaboration, Copenhagen, Denmark).

## Results

As detailed in Figure 1 , 1,281 studies were identified using the search strategy in the three databases and manual search of references of pertinent reviews and meta-analyses. After removal of duplicate articles and unrelated studies, 25 were fully reviewed for the inclusion and exclusion criteria. A total of five studies and 994 patients were included, of whom 502 (50.5%) underwent catheter ablation.^6 , 7 , 10 - 12^Population characteristics are presented in Table 1 . The studies were heterogeneous with regards to the ablation technique, monitoring of recurrent atrial tachyarrhythmias (ATs), and the follow-up period, which ranged from one to five years.

**Figure 1:** – PRISMA flow diagram of study screening and selection

**Table 1:** – Baseline characteristics of included studies

	Number of patients	Male, n(%)	Mean age (years)	Catheter ablation technique	AAD therapy	AT monitoring	Paroxysmal AF, n (%)	Mean time from AF diagnosis (months)	Mean LVEF (%)	Follow-up (years)
RAAFT-1 2005	67	NA	CA: 53 AAD: 54	RF	Flecainide, 77% Sotalol, 23% No AAD reported in ablation group	24-hour Holter before discharge, 3, 6 and 12 months	CA: 32 (97) AAD: 35 (95)	5	CA: 53 AAD: 54	1
MANTRA-PAF 2012	294	CA: 100 (68) AAD: 106 (72)	CA: 56 AAD: 54	RF	Class IC drugs preferred; class III second line; AAD allowed in ablation group for the 3-month blanking period	7-day Holter monitoring at 3, 6, 12, 18 and 24 months	CA: 146 (100) AAD: 148 (100)	NA	LVEF >60%: 237 (80%)	5*
RAAFT-2 2014	127	CA: 51 (77.3) AAD: 45 (73.8)	CA: 56 AAD: 54	RF	During 90-day blanking period: Flecainide, 69%; propafenone 25%; dronedarone 3%. AAD allowed in ablation group	ECG, Holter, transtelephonic monitor, or rhythm strip	CA: 65 (98) AAD: 59 (97)	NA	CA: 61 AAD: 61	2
STOP-AF 2020	203	CA: 63 (61) AAD: 57 (58)	CA: 60 AAD: 62	CB	In AAD group: flecainide 60%; propafenone 7%; dronedarone 12%; sotalol 7%; amiodarone 2%. In ablation group, AAD allowed for 80 days in blanking period.	12-lead ECG conducted at baseline, 1, 3, 6, and 12 months; patient-activated telephone monitoring weekly and when symptomatic at 3-12 months; 24h ambulatory monitoring at 6 and 12 months	CA: 104 (100) AAD: 99 (100)	15.6	CA: 61 AAD: 61	1
EARLY-AF 2020	303	CA: 112 (72.7) AAD: 102 (68.5)	CA: 58 AAD: 59	CB	Flecainide 76%; propafenone 5%; sotalol 15%; dronedarone 3%; AAD allowed in blanking period.	Implantable cardiac monitoring; manual weekly transmissions; visits at 3, 6 and 12 months	CA: 147 (95) AAD: 140 (94)	1	CA: 60 AAD: 60	1

*P values < 0.05 were considered statistically significant in all included studies; ‡hypertension and structural heart disease; AAD: antiarrhythmic drugs; AF: atrial fibrillation; AT: atrial tachyarrhythmia; CA: catheter ablation; CB: cryoablation; ECG; electrocardiogram; LVEF: left ventricular ejection fraction; NA: not available; RF: radiofrequency.*

Recurrence of ATs were significantly less frequent in patients treated with catheter ablation (147/502; 29.2%) as compared with AAD (245/492; 49.8%) (OR 0.36; 95% CI 0.25-0.52; p<0.001; Figure 2 ). Similarly, symptomatic recurrences of AF were also reduced in patients randomized to catheter ablation (57/398; 14.3%) compared with AADs (118/393; 30%) (OR 0.32; 95% CI 0.18-0.57; p<0.001; Figure 3 ). Hospitalizations were also less frequent in the catheter ablation group (21/436; 4.8% vs. 66/431; 15.3%) (OR 0.25; 95% CI 0.15-0.42; p<0.001; Figure 4 ).

**Figure 2:** – Recurrences of atrial tachyarrhythmias were significantly less common with catheter ablation compared to antiarrhythmic drugs (p<0.001). AAD: antiarrhythmic drugs.

**Figure 3:** – Recurrences of symptomatic AF were significantly less common with catheter ablation compared to antiarrhythmic drugs. (p<0.001). AAD: antiarrhythmic drugs.

**Figure 4:** – Hospitalizations were significantly less common with catheter ablation compared to antiarrhythmic drugs (p<0.001). AAD: antiarrhythmic drugs.

Regarding safety endpoints, symptomatic bradycardia (OR 0.55; 95% CI 0.18-1.65; p=0.28; I^2^=0%; Figure 5 ) was not significantly different between patients treated with catheter ablation (3/502; 0.6%) and AAD therapy (7/492; 1.4%). A clinically significant pericardial effusion or pericardial tamponade occurred in 8 of 464 patients in the catheter ablation group (1.7%).

**Figure 5:** – The incidence of symptomatic bradycardia was rare and similar between groups (p=0.28). AAD: antiarrhythmic drugs.

Supplementary Table 1 outlines the quality appraisal of each individual RCT. All studies were considered at risk for performance bias, given the impossibility to perform patient and investigator blinding in the trials. Otherwise, studies were judged to be at low risk of biases. Sensitivity analyses were performed by systematically removing each study from the pooled estimates. After removal of each individual study, the results for recurrences of ATs, symptomatic AF, hospitalizations, and symptomatic bradycardia were unchanged. Although limited by the small number of studies, there was no definitive evidence of publication bias in the funnel plots ( Supplementary Figure 1 ).

## Discussion

In this systematic review and meta-analysis of five studies and 994 patients, we compared catheter ablation with AAD as first-line therapy for rhythm control in patients with AF. The main findings were as follows: (1) the incidence of symptomatic recurrent AF over a follow-up period of one to five years was approximately halved by catheter ablation as compared with AAD (14.3% vs. 30.0%, respectively; OR 0.32; p<0.001); (2) this difference was also statistically significant for reduction of ATs, favoring catheter ablation (OR 0.36; p<0.001); and (3) there was a 3-fold decrease in hospitalizations among those who underwent catheter ablation (4.8% vs. 14.3%).

Catheter ablation has proven to be a superior option to escalation of AAD therapy for rhythm control among patients who have recurrent AF despite an initial attempt of AAD therapy or prior ablation. In the Catheter Ablation vs. Antiarrhythmic Drug Therapy for Atrial Fibrillation (CABANA) trial, 2,204 patients with symptomatic AF with current or past use of ≥1 AAD were randomized to catheter ablation with pulmonary vein isolation or drug therapy. In intention-to-treat analysis, over a median follow-up of 48.5 months, AF recurrence occurred in 49.9% of patients randomized to catheter ablation and 69.5% of those in the drug therapy arm (HR 0.52; 95% CI 0.45-0.60; p<0.001). The primary outcome of death, disabling stroke, serious bleeding or cardiac arrest was not significantly different between ablation and AAD therapy (8% vs. 9.2%, respectively; HR 0.86; p=0.30).^13^

Despite disappointing results with regards to mortality and vascular endpoints, CABANA and other trials unquestionably show a higher efficacy of catheter ablation as compared with AAD therapy alone in patients who previously failed rhythm control with AAD.^13 - 16^Nevertheless, there has been a renewed interest in effective rhythm control early in the natural history of AF. Indeed, the notion that ‘AF begets AF’, due to atrial fibrosis and adverse remodeling, is well-known for nearly three decades.^17 , 18^In the recently published Treatment of Atrial Fibrillation for Stroke Prevention Trial (EAST-AFNET 4), 2,789 patients with AF diagnosed within the prior 12 months (27% persistent AF) were randomized to early rhythm control with catheter ablation (8%) or AAD (87%) or to usual care with rate control and rhythm control for refractory symptoms. Over a median follow-up of 5.1 years, there was a significant reduction in the primary endpoint of cardiovascular death, stroke, or hospitalization with heart failure or acute coronary syndrome favoring early rhythm control (3.9 per 100 person-years) over usual care (5.0 per 100 person-years) (HR 0.79; 96% CI 0.66-0.94; p=0.005).^19^

The strategy of early rhythm control with AAD, however, is limited by reduced efficacy of drug therapy alone. A recent systematic review and meta-analysis from the Cochrane Collaboration examined the efficacy and safety of AADs in 59 RCTs with 20,981 participants, including both paroxysmal and persistent AF. Over a mean follow-up of 10.2 months, AF recurred in 43-67% of patients treated with AADs.^20^The limited efficacy of AAD therapy is quite evident when considering the high cross-over rate from the AAD arm to the catheter ablation arm in randomized trials. In the STOP AF First: Cryoballoon Catheter Ablation in Antiarrhythmic Drug Naïve Paroxysmal Atrial Fibrillation trial, a third of patients in the AAD group underwent an ablation due to drug therapy side effect or recurrence of AF.^6^In the CABANA trial, 27.5% of patients in the AAD group crossed over to catheter ablation during follow-up.^13^

Earlier trials comparing the efficacy of catheter ablation to AAD therapy in patients naïve to any rhythm control strategy were limited by small sample sizes.^10 , 12^Collectively, these studies did not determine a conclusive superiority of catheter ablation over AAD therapy.^10 - 12^A meta-analysis of these trials found a significantly lower freedom from AF recurrence with catheter ablation, relative to AAD therapy (risk ratio [RR] 0.63; 95% CI 0.44-0.92; p=0.02); however, the rate of symptomatic AF recurrences was not significantly different between groups (RR 0.57; 95% CI 0.30-1.08; p=0.09).^21^Therefore, the STOP AF First and the Cryoablation or Drug Therapy for Initial Treatment of Atrial Fibrillation (EARLY-AF) trials were designed to further investigate the role of catheter ablation as a first-line rhythm control strategy.

Our findings provide a more accurate understanding of the treatment effect by pooling a large population of patients randomized to catheter ablation or AAD therapy. The magnitude of effect favoring catheter ablation was substantial. The absolute reduction in the frequency of AT and symptomatic AF with catheter ablation as compared with AAD therapy was 20% and 15%, respectively. When considering these findings, safety outcomes of catheter ablation must not be overlooked, to guide shared decision-making. The pooled incidence of significant pericardial effusions and/or pericardial tamponade in these studies was 1.7%. In a meta-analysis of nearly 9,000 patients who underwent cryoablation or radiofrequency ablation, the incidence of pericardial tamponade was 1.1%. Phrenic nerve palsy occurred in 1.6% of patients who underwent cryoablation, but the vast majority resolved during short-term follow-up.^22^

As shown in Table 1 , the ablation techniques were heterogeneous between studies. The three earlier studies used radiofrequency ablation, whereas the more recent STOP-AF and EARLY-AF trial used cryoballoon.^6 , 7 , 10 - 12^Although the techniques have important differences in operator learning curves and safety endpoints, the FIRE and ICE randomized trial^23^and a meta-analysis^22^have shown similar efficacy between the two techniques. More importantly, radiofrequency technology has improved substantially in recent years, particularly with the development of contact force sensors, which were not used in the radiofrequency trials included in the present study. A meta-analysis of 22 studies showed that contact force-guided catheter ablation substantially reduced procedure time and improved AF-free survival by 12%.^24^Whether the use of newer technology for radiofrequency catheter ablation would modify the comparative efficacy of catheter ablation vs. AAD for initial rhythm control in symptomatic AF is unknown. However, if so, this would translate into an even more favorable effect of ablation relative to AAD therapy.

Our study has limitations. First, long-term follow-up beyond two years was only possible for two out of five studies. Second, the rhythm monitoring strategy was heterogeneous between studies as outlined in Table 1 , varying from periodic Holter monitoring to continuous cardiac monitoring. However, sensitivity analysis removing one study at a time did not alter the significance of efficacy estimates. Third, the absence of patient-level data precluded more granular assessment of outcomes, such as time-to-recurrence of AT/AF. Finally, the small number of studies did not allow for subgroup analyses of different catheter ablation techniques. However, a prior meta-analysis has shown similar efficacy between radiofrequency and cryoballoon ablation.^22^

## Conclusion

In summary, catheter ablation significantly reduces the recurrence of AT and symptomatic AF as compared with AAD therapy in patients who are naïve to prior attempts of rhythm control. This study provides evidence supporting catheter ablation as a class I indication for rhythm control in patients with paroxysmal AF.

### Data sharing agreement

Because this meta-analysis was based on data extracted from previously published research, all data and study materials are available in the public domain. The authors of this meta-analysis did not have access to patient-level data of the included studies, and researchers interested in these data are encouraged to contact the corresponding authors of each study.

## * Supplemental Materials

For additional information, please click here.
